# Evaluating [^11^C]PBR28 PET for Monitoring Gut and Brain Inflammation in a Rat Model of Chemically Induced Colitis

**DOI:** 10.1007/s11307-016-0979-0

**Published:** 2016-07-11

**Authors:** E. Kurtys, J. Doorduin, U. L. M. Eisel, R. A. J. O. Dierckx, E. F. J. de Vries

**Affiliations:** 1 Department of Nuclear Medicine and Molecular Imaging, University of Groningen, University Medical Center Groningen, PO Box 30.001, 9700 RB Groningen, The Netherlands; 2 Department of Molecular Neurobiology, Center for Life Sciences, University of Groningen, Groningen, The Netherlands

**Keywords:** Macrophage, Colitis, 2,4,6-Trinitrobenzenesulfonic acid, Neuroinflammation, PET, Imaging

## Abstract

**Purpose:**

Ulcerative colitis (UC) is a chronic inflammatory disease of the colon that affects an increasing number of patients. High comorbidity is observed between UC and other diseases in which inflammation may be involved, including brain diseases such as cognitive impairment, mental disorders, anxiety, and depression. To investigate the increased occurrence of these brain diseases in patients with UC, non-invasive methods for monitoring peripheral and central inflammation could be applied. Therefore, the goal of this study is to assess the feasibility of monitoring gut and brain inflammation in a rat model of chemically induced colitis by positron emission tomography (PET) with [^11^C]PBR28, a tracer targeting the translocator protein (TSPO), which is upregulated when microglia and macrophages are activated.

**Procedures:**

Colitis was induced in rats by intra-rectal injection of 2,4,6-trinitrobenzenesulfonic acid (TNBS). Rats with colitis and healthy control animals were subjected to [^11^C]PBR28 PET of the abdomen followed by *ex vivo* biodistribution in order to assess whether inflammation in the gut could be detected. Another group of rats with colitis underwent repetitive [^11^C]PBR28 PET imaging of the brain to investigate the development of neuroinflammation.

**Results:**

Eleven days after TNBS injection, *ex vivo* biodistribution studies demonstrated increased [^11^C]PBR28 uptake in the inflamed cecum and colon of rats with colitis as compared to healthy controls, whereas PET imaging did not show any difference between groups at any time. Similarly, repetitive PET imaging of the brain did not reveal any neuroinflammation induced by the TNBS administration in the colon. In contrast, significantly increased [^11^C]PBR28 uptake in cerebellum could be detected in *ex vivo* biodistribution studies on day 11.

**Conclusion:**

Inflammation in both the gut and the brain of rats with chemically induced colitis was observed by *ex vivo* biodistribution. However, these effects could not be detected by [^11^C]PBR28 PET imaging in our colitis model, which is likely due to spill-over effects and insufficient resolution of the PET camera.

## Introduction

Ulcerative colitis (UC) and Crohn’s disease are the major types of inflammatory bowel disease (IBD). UC is a chronic disease that is thought to be caused by a pathological inflammatory response to intestinal microbes in genetically susceptible patients [1]. UC is characterized by strong inflammation in the colon and rectum that leads to diarrhea, cramping, and pain [2]. The pathogenesis of IBD is still poorly understood, and consequently, an effective treatment is not available yet [3, 4]. High comorbidity between IBD and brain diseases, including cognitive impairment [5], mental disorders [6], anxiety, and depression [7–9], is observed. Increasing evidence suggests that inflammation plays a role in the development of these brain diseases [10–13], and as a result, it is hypothesized that the comorbidity between IBD and, e.g., depression is mediated by inflammation via gut–brain interactions [14].

Evidence suggests that colitis can induce neuroinflammation and affect brain functions. For example, in animals, induction of colitis caused an increase in pro-inflammatory cytokines, reduced neurogenesis in hippocampus [15] and behavioral abnormalities such as cognition impairment, and depression- and anxiety-like behavior which persisted after the colonic inflammation had resolved [16, 17]. In humans, the gut-brain interaction during colitis has been suggested to be responsible for inflammation-mediated changes in gray matter in patients with IBD [18]. Several lines of evidence suggest that inflammation could be a promising target for the treatment of colitis-related brain disorders such as depression [19] or cognitive decline [20]. In fact, immunosuppressive treatment was shown to ameliorate depressive symptoms in patients with IBD [21].

In the clinic, the best way to diagnose colitis is by colonoscopy. This method, however, is not suitable for investigating the comorbidity of IBD. Non-invasive methods can allow investigation of inflammation in peripheral tissues and, more importantly, in the brain. Thus, adequate imaging methods could help to increase our understanding of the psychiatric comorbidities associated with colitis.

Positron emission tomography (PET) is a powerful tool for non-invasive detection of inflammation both in animals and in patients. So far, intestinal inflammation in humans and mice was mainly assessed with PET imaging of glucose metabolism with 2-deoxy-2-[^18^F]fluoro-D-glucose ([^18^F]FDG) [22–25]. This method, however, has some limitations, as physiological [^18^F]FDG uptake can be found in some patients [26]. Moreover, [^18^F]FDG uptake is not specific for inflammation and it can be affected by various factors, including stress and physical activity. Therefore, more specific approaches with tracers targeting specific inflammatory markers might be a good alternative for [^18^F]FDG PET.

Colitis is characterized by massive infiltration of activated macrophages and other immune cells in the affected colon. In the mitochondrial membrane of activated macrophages, the expression of 18-kDa translocator protein (TSPO) receptors is strongly increased [27, 28]. In fact, high levels of TSPO receptors were observed in the colon of patients with IBD and in animal models of colitis [29, 40]. Therefore, the TSPO receptor could be a suitable biomarker to monitor colitis-related intestinal inflammation. TSPO receptors have already been used as an imaging biomarker for neuroinflammation. [^11^C]PBR28 ([O-methyl-11C] N-acetyl-N-(2-methoxybenzyl)-2-phenoxy-5-pyridinamine) was developed as a tracer for PET imaging of TSPO expression in the brain. Based on biomathematical modeling approaches, [^11^C]PBR28 PET demonstrated relatively low test–retest variability in repeated measurements in the same subject, high specific binding to TSPO receptors, and a good signal-to-noise ratio as compared to the other TSPO tracers [30]. We, therefore, selected [^11^C]PBR28 PET to investigate whether gut and brain inflammation associated with colitis could be detected in an animal model of chemically induced colitis.

Several chemically induced animal models are used to mimic UC in humans. Colitis induced with 2,4,6-trinitrobenzenesulfonic acid (TNBS) is a widely used model to monitor temporal changes in inflammatory markers and other symptoms characteristic for UC. Intracolonic TNBS administration causes chemically induced damage of the intestinal mucosa, leading to the development of the characteristic symptoms observed in UC patients, such as diarrhea and presence of blood in the feces [31]. TNBS-induced colitis in rats has also been used to study effects of gut inflammation on the brain. TNBS administration in rats was shown to induce activation of microglia and cytokine release in the brain [32, 33] and increased expression of intestinal TSPO receptors [40]. Therefore, we selected the TNBS-induced colitis model for this study. The purpose of the study was to investigate whether *in vivo* detection of the activation of macrophages/microglia in the experimental model of colitis with PBR28 PET is feasible.

## Material and Methods

### Experimental Animals

Animal experiments were performed in accordance with Dutch Regulations for Animal Welfare. All procedures were approved by the Institutional Animal Care and Use Committee of the University of Groningen (protocol DEC6576D).

Male outbred Sprague Dawley rats (7 weeks of age, *n* = 24) were purchased from Harlan (Horst, The Netherlands). Animals were housed in thermo-regulated (21 ± 2 °C) and humidity-controlled rooms under a 12–12 h light–dark cycle (lights on at 6:00 am) with ad libitum access to food (standard laboratory chow, AIN93-G) and water. After arrival, the rats were housed in groups and allowed to acclimatize for at least 7 days. Animals were single housed during the experiment to allow individual measurement of food intake and assessment of colitis symptoms.

### Study Design

This study was divided into two parts. First, the abdominal imaging followed by *ex vivo* biodistribution was performed. In the second part, longitudinal brain imaging was performed. The time points for PET and *ex vivo* biodistribution were selected on day 4 and day 11 after TNBS administration based on literature data [32]. Day 4 represents the peak of inflammation in the gut, whereas inflammation was expected to be resolved by day 11.

**Fig. 1:**
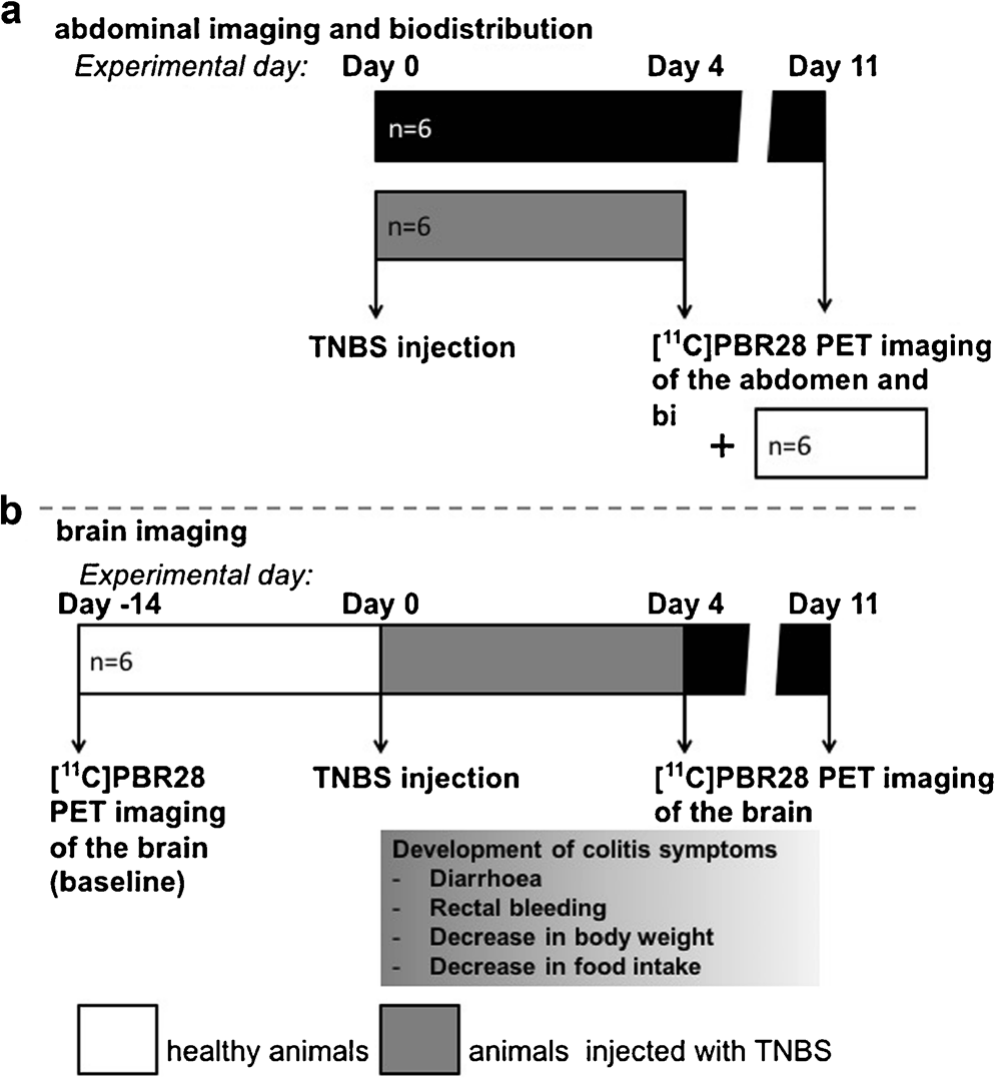
Study design. **a** Three experimental groups were injected with [^11^C]PBR28, subjected to PET imaging of the abdomen and sacrificed for *ex vivo* biodistribution to determine standardized uptake values (SUV) in various organs and tissues. The healthy control group (*n* = 6, *white*) was terminated at day 4. 2,4,6-Trinitrobenzenesulfonic acid (TNBS) [30 mg/rat]-treated rats were terminated at either at day 4 (*n* = 6, *gray*) or day 11 (*n* = 6, *black*). **b** A group of rats (*n* = 6) was investigated by PET imaging of the brain 14 days before TNBS injection (baseline) and 4 and 11 days after TNBS injection.

In the first part of the study, rats were randomly divided into three groups (*n* = 6 per group): (1) controls, (2) TNBS-injected rats sacrificed on day 4 post-injection, and (3) TNBS-injected rats sacrificed on day 11 post-injection (Fig. 1a). Rats received an intra-rectal injection of TNBS under isoflurane anesthesia on day 0, and disease progression was monitored daily. Rats were injected with [^11^C]PBR28, subjected to a 60-min PET scan of the abdomen, and sacrificed approximately 65 min after the tracer injection. This was followed by isolation of organs and tissues (identified in Figs. 3f, 4, and 5a) for *ex vivo* biodistribution.

In the second part of the study, six healthy rats were subjected to a baseline [^11^C]PBR28 PET scan of the brain (Fig. 1b). Two weeks after the baseline PET scan, on experimental day 0, these animals (*n* = 6) were intra-rectally injected with TNBS under isoflurane anesthesia (see below). Further, rats were subjected to a [^11^C]PBR28 PET scan of brain on day 4 and 11. Disease progression was monitored during the whole period after TNBS injection.

### TNBS Injection

The rat model of TNBS-induced colitis was employed according to a previously described method [32, 34]. Briefly, on experimental day 0, rats were anesthetized with isoflurane (5 % induction and 3 % maintenance) mixed with oxygen. The abdomen was elevated at approximately 45° and TNBS (30 mg/rat, in 1:1 [vol/vol] mixture of absolute ethanol and saline) was injected into the colon via a cannula which was carefully inserted into the rectum (approximately 8 cm from the anus). The abdomen remained elevated at approximately 45° for 30 min to avoid leakage of TNBS. The body temperature of the rats was maintained with heating pads; eye salve was applied onto the eyes to prevent dehydration. Control animals were not subjected to any procedure on experimental day 0 to avoid incidental inflammation due to mechanical damage of the gut mucosa. After rectal TNBS administration, rats were single housed, and body weight, food intake, and feces condition were monitored daily. A 4-point scale was applied to assess feces conditions: score 0 for normal feces, 1 for soft feces with a normal form, 2 for diarrhea, and 3 for the absence of feces production.

### PET Imaging

[^11^C]PBR28 with a radiochemical purity >99 % and a specific activity of 210 ± 15 GBq/μmol were synthesized as previously described [35]. The specific activity of [^11^C]PBR28 was not significantly different between the experimental groups (*F*(3) = 0.944, *p* = 0.439). PET scans were performed using a dedicated small animal PET scanner (MicroPET Focus 220, Siemens Medical Solutions, USA) in healthy animals or at day 4 or 11 after TNBS injection. Two rats from different experimental groups were scanned simultaneously in each scanning session. The body temperature of the rats was maintained with heating pads, eye salve was applied onto the eyes to prevent dehydration, and heart rate and blood oxygen levels were monitored with BioVet system (M2 M Imaging, USA).

For abdominal PET imaging, rats were anesthetized with isoflurane mixed with medical air (5 % induction and 3 % maintenance) and a cannula was inserted into the tail vein for the injection of the PET tracer. Rats were positioned in the PET camera in a transaxial position with their abdomen in the center of the field of view. A transmission scan of 10 min with a Co-57 point source was performed and used to correct the PET data for attenuation, scatter and random coincidences, and decay of radioactivity. After completion of the transmission scan, [^11^C]PBR28 (29 ± 11 MBq) was injected over a 1-min period and a 60-min emission scan was started. There was no significant difference in injected [^11^C]PBR28 dose between the groups (*F* = 1.558, *p* = 0.254). Rats were sacrificed after acquisition of the emission scan and tissues were collected for *ex vivo* biodistribution.

To investigate whether microglia were activated as a results of colitis induction, animals were subjected to PET imaging of the brain at three time points. The scans were performed in the same way as for the abdomen, except for the positioning of the animals in the PET camera. Rats were positioned with their heads in the center of the field of view. A bolus of 21 ± 9 MBq [^11^C]PBR28 was injected, with no statistically significant difference in injected dose between time points (*F* = 0.173, *p* = 0.843). After the scan, rats were allowed to recover from anesthesia in their home cage, which was placed on a heating pad for at least 2 h.

### PET Image Reconstruction and Analysis

List-mode data of the [^11^C]PBR28 PET scans was separated into 21 frames (6 × 10, 4 × 30, 2 × 60, 1 × 120, 1 × 180, 4 × 300, and 3 × 600 s). Emission sinograms were iteratively reconstructed using OSEM2D (4 iterations and 16 subsets). The [^11^C]PBR28 PET scans of the abdomen were analyzed with Vinci 4.26 software (Max Planck Institute for Neurological Research, Germany). Volumes of interest (VOIs) of the abdomen were drawn manually. In the exploratory analysis, VOIs of several sizes were drawn, giving similar results. Final analysis was done with VOIs of 7015 mm^3^ (9,784 voxels). The [^11^C]PBR28 PET scans of the brain were automatically co-registered with a tracer-specific template [36] and spatially aligned with a stereotaxic T2-weigthed MRI template in Paxinos space [37] using Vinci 4.26 software. For both abdominal and brain imaging, a frame of 10 min, starting 50 min post-injection, was chosen for analysis, as this time frame has been shown to be the most stable in recent studies with [^11^C]PBR28 PET [38]. Standardized uptake value (SUV) of the tracer for VOIs in the abdomen and the brain were calculated as follows: [tissue activity concentration (MBq/ml) × body weight (g)]/[injected dose (MBq)].

### *Ex Vivo* Biodistribution

Control and experimental animals were sacrificed after abdominal PET imaging, and the uptake of [^11^C]PBR28 in various tissues identified was measured *ex vivo*. Samples from major organs, bowels, and brain were collected and weighted. Radioactivity in the tissues was measured with a gamma counter (LKB Wallace, Finland). The results of biodistribution studies are expressed as SUV according to the following equation: [tissue activity concentration (MBq/ml) × body weight (g)]/[injected dose (MBq)], which was calculated as indicated in the previous section. The same scale (SUV) was used for both imaging and *ex vivo* biodistribution in order to facilitate comparison between both measurements.

### Statistical Analysis

Results are presented as mean ± standard deviation. Differences in body weight, injected dose, and specific activity of [^11^C]PBR28 between the experimental groups were analyzed by one-way ANOVA.

SUVs obtained from imaging and biodistribution studies were analyzed by one-way ANOVA, followed by a Dunnett post hoc test with the control groups as reference. A probability (*p*) value <0.05 was considered as statistically significant.

## Results

### Disease Symptoms

**Fig. 2:**
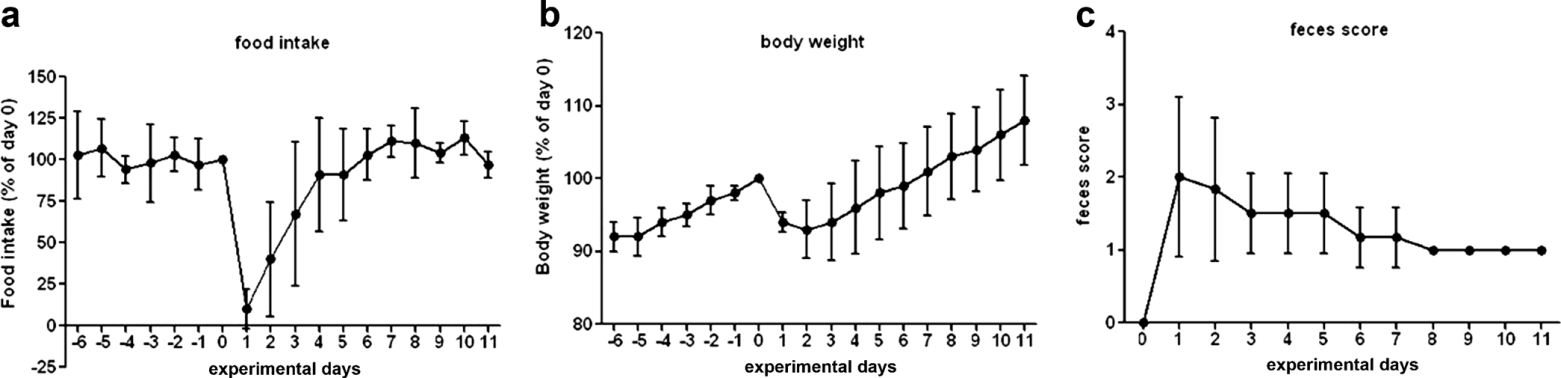
Disease progression. Influence of colitis induced by intra-rectal injection of TNBS at day 0 on **a** food intake, **b** body weight, and **c** feces score as a function of time. Fecal scored 0 = normal feces, 1 = soft feces, 2 = diarrhea, and 3 = no fecal production. Data for food intake and body weight in healthy controls are represented by the values from day −6 to 0, before TNBS injection. For the fecal score, no symptoms of colitis were observed in healthy animals (score 0). After day 7 following TNBS injection, the feces score was 1 for all animals (therefore, no error bar is displayed). Data are presented as mean ± standard deviation (*n* = 6). The animals did not undergo any other interventions or procedures within the displayed time frame.

Rectal administration of TNBS resulted in the development of clinical symptoms of colitis, which manifested as a decrease in food intake and a decrease in body weight, similar to what has been observed in previous studies [33, 34, 39]. On the experimental day 0, the body weight was 352 ± 24 g and did not differ significantly between the groups (one-way ANOVA, *F* = 0.512, *p* = 0.61). As shown in Fig. 2, food intake on day 1, post-TNBS injection, was reduced by 90 ± 12 % (2.2 ± 2.6 g versus 21 ± 2 g, *F* = 160.0, *p* < 0.0001). As a result, body weight was reduced by 19 ± 4 g on day 2 after TNBS injection (6 % ± 1 % as compared to day 0). By day 7, body weight and food intake had recovered to baseline levels again. Diarrhea was observed in all rats after TNBS treatment. Some rats did not produce any feces for up to 2 days following TNBS administration. In some animals, blood in the feces and fecal adhesion to the anus was noted. The consistency of the feces remained abnormal until sacrifice on day 11 post-TNBS administration, indicating that the animals had not fully recovered at the end of the experiment. This was confirmed by the abnormal appearance of the excised colon.

### *Ex Vivo* Biodistribution and PET Imaging of the Abdomen

**Fig. 3:**
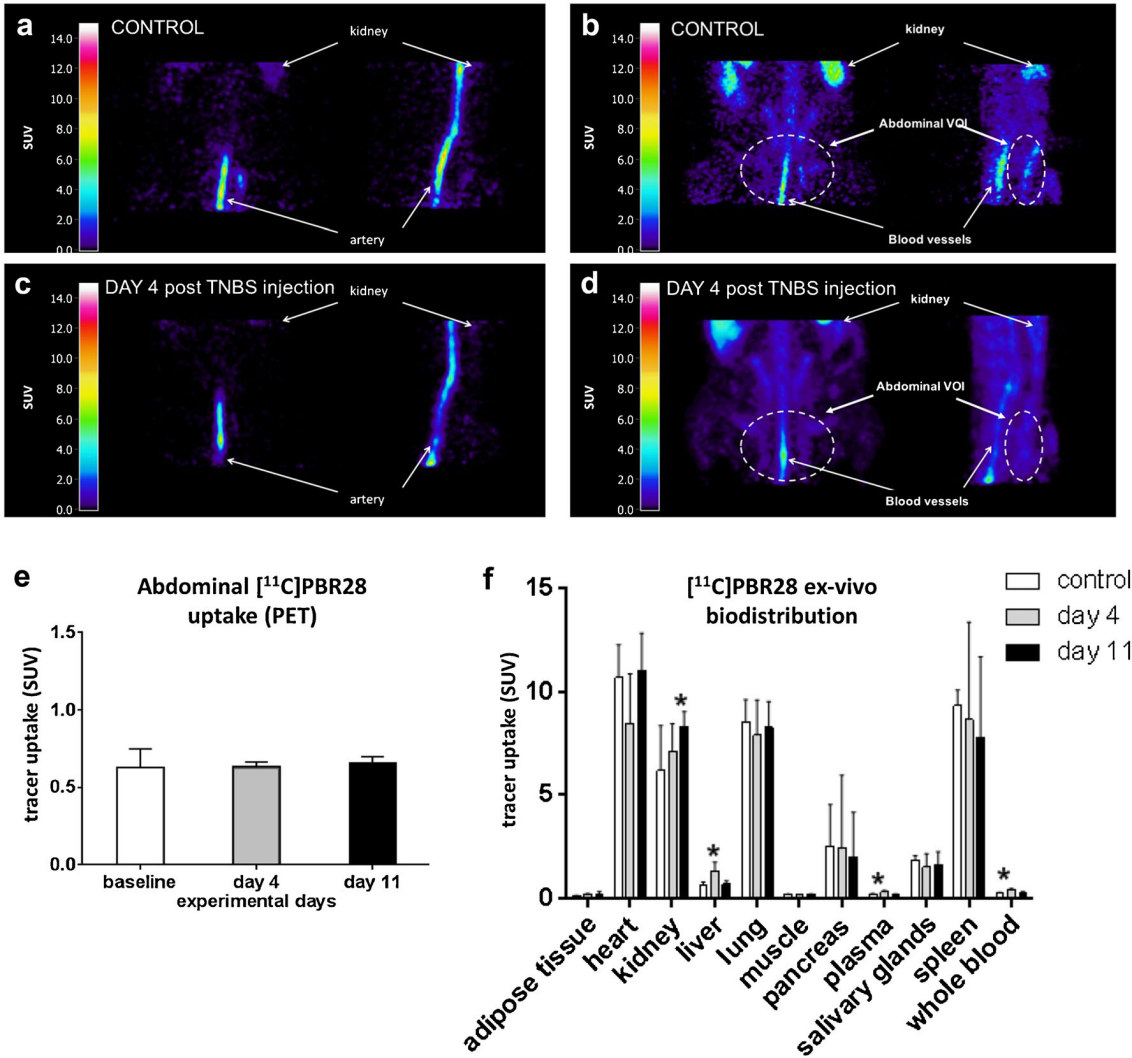
PET imaging and *ex vivo* biodistribution of peripheral organs and tissues as a function of disease progression. Healthy rats or TNBS-treated rats at either day 4 or day 11 post-TNBS injection were injected intravenously with [^11^C]PBR28 (29 ± 11 MBq) and subjected to the a 60-min PET scan of the abdomen under isoflurane anesthesia: examples of sagittal and coronal sections showing the **a** first 50 s of the scan and **b** the last 10 min of the scan of a control rat; **c** the first 50 s of the scan and **d** the last 10 min of the scan of a rat 4 days after TNBS injection; **e** abdominal uptake (SUV) of [^11^C]PBR28 [mean ± standard deviation] in TNBS-treated and control rats. Immediately after the PET scan (65 min after tracer injection), animals were euthanized by cardiac puncture under deep sevoflurane anesthesia. Organs and tissues were harvested for *ex vivo* biodistribution. [^11^C]PBR28 uptake in major organs and tissues as determined by **f** *ex vivo* biodistribution (mean ± standard deviation). Statistically significant differences between TNBS-treated animals and the control group are indicated with an *asterisk*: *p < 0.05 (ANOVA with Dunnett post hoc test).

As shown in Fig. 3, abdominal [^11^C]PBR28 PET images of control rats and rats treated with TNBS show a similar tracer distribution. An unknown hotspot in the abdomen was observed in all investigated groups (Fig. 3). By analyzing different time frames of the PET scan, we observed that this unknown hotspot was already clearly visible during the first 50 s of the scan (i.e., during the tracer injection), when the tracer is supposed to be mainly present in the blood (Fig. 3a, c). This implies that the observed hotspots are not representing the (inflamed) colon, but probably represent the blood vessels. The analysis of later frames showed that hotspots located in blood vessels remain clearly visible until the end of the 60 min scan and therefore the blood vessels could be a potential pitfall when interpreting [^11^C]PBR28 PET images of the abdomen. On the images acquired between 50 and 60 min after tracer injection, the highest uptake is observed in the kidneys and in the blood vessels, both in controls (Fig. 3b) and in TNBS-treated animals (Fig. 3d). The inflamed colon could not be clearly discriminated, and therefore, the whole abdomen was analyzed. [^11^C]PBR28 uptake in the abdomen (excluding kidney and visible blood vessels from the volume of interest) did not reveal any differences between the groups (control: SUV 0.62 ± 0.12, day 4 after TNBS injection: SUV 0.63 ± 0.03, day 11 after TNBS injection: 0.66 ± 0.04; *F* = 0.288, *p* = 0.76).

**Fig. 4:**
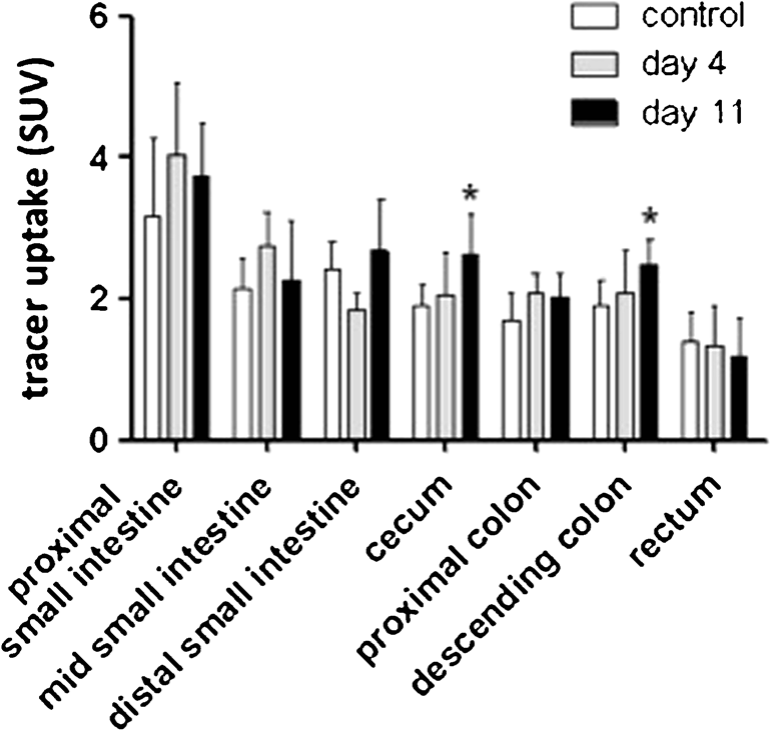
*Ex vivo* Biodistribution of the intestines as a function of disease progression. [^11^C]PRB28 uptake (60 min) in the intestines of control rats (*n* = 6) and TNBS-treated rats sacrificed at day 4 (*n* = 6) and day 11 (*n* = 6). Data are presented as mean ± standard deviation. Statistically significant differences between TNBS-treated animals and the control group are indicated with an *asterisk*: *p < 0.05 (ANOVA with Dunnett post hoc test).

Investigation of the intestinal tract by *ex vivo* biodistribution on day 4 post-TNBS injection did not reveal any significant differences between controls and rats with colitis either. On day 11, however, significantly higher [^11^C]PBR28 uptake was observed in cecum and colon (2.23 ± 0.14 vs. 1.80 ± 0.28, *p* = 0.02) of rats from the colitis group as compared to controls (Fig. 4).

*Ex vivo* biodistribution studies of tissues beyond other than the gastrointestinal tract at day 4 post-TNBS injection demonstrated that radioactivity concentrations in plasma (SUV 0.30 ± 0.08 vs. 0.20 ± 0.03, *p* < 0.01), whole blood (SUV 0.39 ± 0.08 vs. 0.25 ± 0.04, *p* < 0.001), and liver (SUV 1.31 ± 0.45 vs. 0.65 ± 0.14, *p* = 0.001) were significantly higher in rats from the colitis group than in controls. At day 11 post-TNBS injections, rats from the colitis group had significantly higher tracer uptake in kidneys than controls (SUV 8.30 ± 0.70 vs. 6.20 ± 2.20, *p* = 0.024).

### *Ex Vivo* Biodistribution and PET Imaging of the Brain

**Fig. 5:**
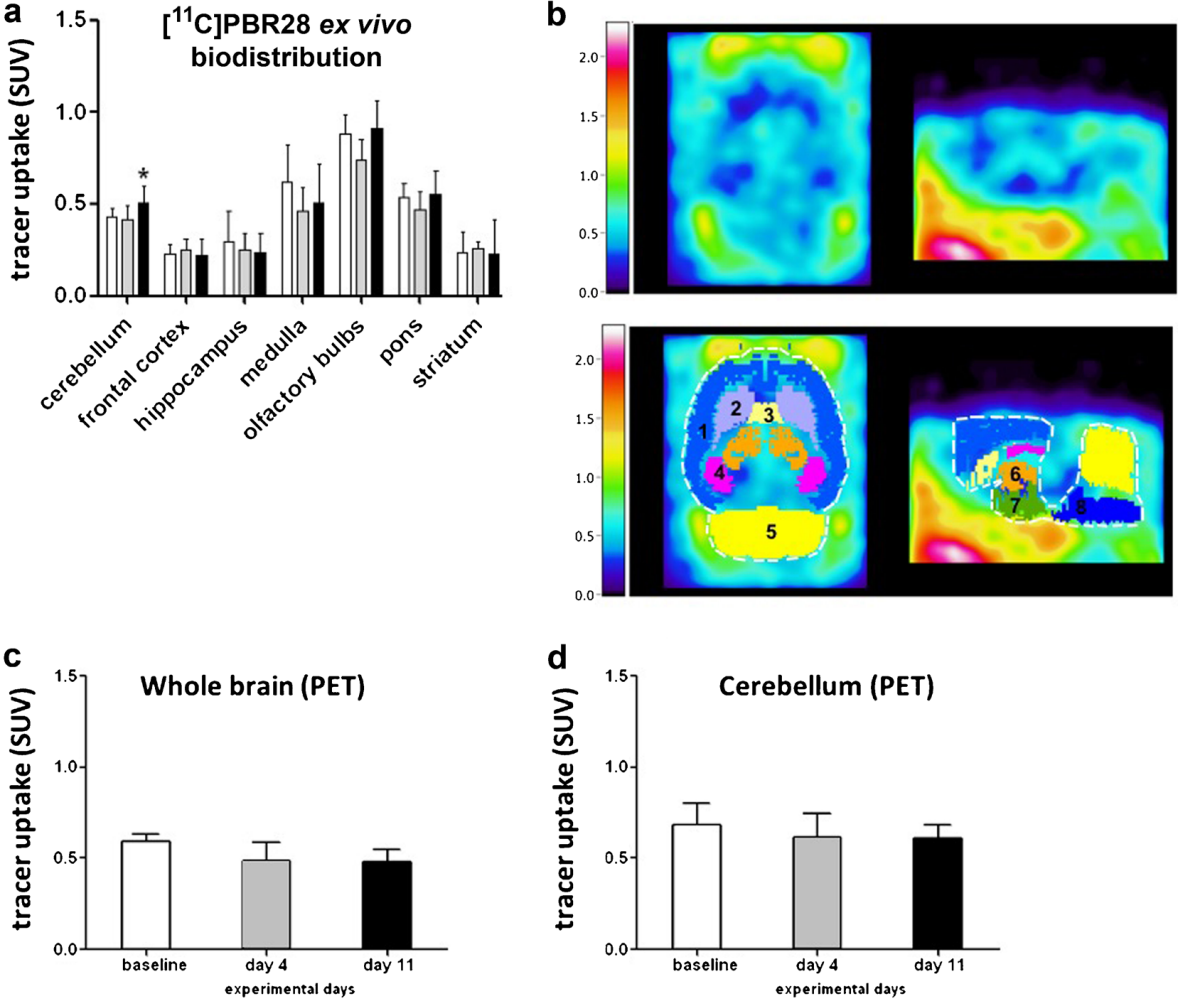
*Ex vivo* biodistribution and PET imaging of the brain. **a** *Ex vivo* biodistribution of [^11^C]PRB28 (65 min) in the brains of control rats (*n* = 6) and TNBS-treated rats sacrificed at day 4 (*n* = 6) and day 11 (*n* = 6). **b** An example of a sagittal and coronal [^11^C]PRB28 PET image of the brain of a rat subjected to TNBS treatment (day 4) and its overlay with a MRI template with selected regions, as indicated by different colors and the numbers: *1* = cortex, *2* = caudate putamen, *3* = septum, *4* = hippocampus, *5* = cerebellum, *6* = thalamus, *7* = hypothalamus, *8* = pons + medulla. [^11^C]PRB28 uptake (50–60 min) in **c** whole brain and **d** cerebellum was measured by PET imaging. Data are presented as mean ± standard deviation. Statistically significant differences between TNBS-treated animals and the control group are indicated with an *asterisk*: *p < 0.05 (ANOVA with Dunett post hoc test).

As shown in Fig. 5a, *ex vivo* biodistribution showed no significant differences in tracer uptake in the brain between groups on day 4 post-TNBS injection. On day 11, however, significantly higher tracer uptake in the cerebellum of TNBS-treated animals than in controls was found (SUV 0.51 ± 0.09 vs. 0.43 ± 0.05, *p* = 0.043).

In contrast, PET imaging did not show any significant differences in tracer uptake in cerebellum (*F* = 0.648, *p* = 0.54) or whole brain (*F* = 1.069, *p* = 0.37) at any time point after TNBS administration (Figs. 4b and 5c, d), indicating that PET imaging was not sensitive enough to detect the difference in TSPO expression observed in *ex vivo* biodistribution studies.

## Discussion

The goal of the present study was to investigate the feasibility of detecting TNBS-induced inflammation in the gut and the brain of rats using PET imaging with the TSPO-targeting tracer [^11^C]PBR28. In humans, IBD is characterized by a strong peripheral inflammatory response. Previous studies have shown that TNBS administration in the rat colon causes symptoms similar to colitis in humans, such as temporal diarrhea, rectal bleeding, decreased food intake, and loss of body weight. These symptoms were also observed in this study, indicating that colitis was indeed present in the TNBS-treated rats.

Colitis involves inflammation of the gut and is accompanied by infiltration of immune cells, including macrophages, in the colon. We observed that TNBS injection caused an increase in [^11^C]PBR28 uptake in cecum and the descending colon. This would suggest that infiltrating macrophages with elevated TSPO expression are present in these parts on the intestinal tract. This observation is in agreement with studies showing elevated TSPO expression in biopsies from patients with chronic IBD and in tissue sections from the descending colon of animals subjected to chemically induced colitis [29, 40].

Despite the elevated [^11^C]PBR28 uptake in the inflamed intestines, no significant differences in intestinal uptake between rats with colitis and controls could be detected by PET imaging. PET images of the abdomen showed high uptake of [^11^C]PBR28 in kidney and an unknown hotspot in the abdomen, which was initially interpreted as being the inflamed colon, due to the lack of any anatomical reference. However, further investigation of the kinetics of the tracer uptake demonstrated that the hotspot in the abdomen did not represent the inflamed intestine, but the blood vessels. The proximity of blood vessels to the colon may have prevented detection of the inflamed colon by [^11^C]PBR28 PET. The tracer uptake in the blood vessels is approximately threefold higher than in the inflamed colon, and consequently, partial volume and spill-over effects may have obscured the colon on the PET images. *Ex vivo* biodistribution studies, on the other hand, allow separation of the organs that are close together in the body, and therefore, differences in [^11^C]PBR28 uptake between groups could be detected by this method. The differences in SUV values in cecum and descending colon were significant but seem to be minor. Studies on brain demonstrated high specificity of [^11^C]PBR28 for TSPO receptors [40]. We hypothesize that this minor effect could be due to relatively low levels of TSPO receptor expression in rat colitis or due the fact that basal [^11^C]PBR28 in the intestine is relatively high (it is approximately five times higher than background uptake in healthy brain, as measured with *ex vivo* biodistribution). For that reason, it might be difficult to observe any effect of inflammation.

It is important to notice that SUV values for the intestines obtained from *ex vivo* biodistribution are approximately threefold higher than those obtained by PET imaging. This can be explained by the fact that in *ex vivo* biodistribution feces, fluids, and gases inside the intestine are removed, and therefore, do not contribute to the SUV. In PET imaging, on the other hand, a region of interest is drawn around a section of the gut, which includes not only the intestinal wall, but also the contents of the intestine in the intraperitoneal space. It seems likely that the concentration of activated macrophages is primarily located in the intestinal wall. Consequently, the radioactivity signal that originates from the activated macrophages in the colitis is diluted with the radioactivity signal from feces, intestinal lumen, and intraperitoneal space, resulting in a lower SUV for the total region of interest. This cannot be avoided since the spatial resolution of the PET camera is insufficient to draw a region of interest around the intestinal wall alone.

In contrast to our results, other investigators using [^18^F]DPA-714 as the PET tracer reported that the inflamed colon could be visualized by PET in TNBS-injected animals. They described significantly increased tracer uptake in the colon of TNBS-injected rats 7 days after colitis induction, as compared to healthy controls [41]. Remarkably, from the DSS-induced model of colitis used in the same study for *ex vivo* biodistribution, the reported absolute uptake of [^18^F]DPA-714 in the colon as determined by PET was at least an order of magnitude higher than the reported colon uptake of [^18^F]DPA-714 as measured by *ex vivo* biodistribution. This suggests that [^18^F]DPA-714 PET data in the colon may have been affected by a substantial spill-over effect as well, possibly from the blood vessels or the intestinal contents. In the future studies, PET should ideally be combined with simultaneous measurement of contrast-enhanced CT or MRI in order to correctly localize the colon and thus to allow better detection of differences in tracer uptake between conditions. Moreover, localization of the inflamed colon and reducing spill-over from the blood vessels could also be facilitated by injecting TNBS in the proximal colon, rather than in the distal colon, as demonstrated in previous studies [41].

When comparing the *ex vivo* biodistribution results of our study with the reported data for [^18^F]DPA-714 in DSS-colitis model [41], [^11^C]PBR28 uptake in the colon of TNBS-treated rats was approximately tenfold higher than [^18^F]DPA-714 uptake. However, the reported relative difference between the colitis group and controls is much larger for [^18^F]DPA-714 than the difference that we observed for [^11^C]PBR28. This suggests that the relatively high background uptake of [^11^C]PBR28 in the healthy gut may have led to insufficient contrast to detect colitis in rats. However, comparison between the studies should be interpreted with care, since not only different PET tracers were used, but also different time points after TNBS injection were investigated. Therefore, disease severity (i.e., inflammation) may have been different between the two studies.

TNBS administration did not only affect [^11^C]PBR28 uptake in the intestines, but also in liver, plasma, whole blood (day 4), and kidneys (day 11). This is consistent with the previous studies demonstrating the distribution of [^11^C]PBR across the organs and its metabolic fate (renal clearance) [42]. The increase in [^11^C]PBR28 retention in the blood probably reflects the binding of the tracer to immune cells that were activated as a result of the experimental colitis. Since the blood content in the liver is high (approximately 30 %), elevated blood radioactivity levels could also explain the increased tracer uptake in the liver. Moreover, the increased tracer uptake in the liver could also be due to distinct changes in the dendritic cell and macrophage composition in the liver as a result of acute and chronic intestinal inflammation [43]. Previous studies have also shown crosstalk between the gut and kidney during colitis [44, 45]. Chemically induced colitis generates inflammatory mediators that are responsible for neutrophil infiltration in the kidney, resulting in acute kidney injury. Anti-inflammatory agents or knock-out of chemokine receptors could suppress the colitis-induced kidney injury [44, 45]. Thus, infiltration of activated macrophages may have been responsible for the elevated tracer uptake in the kidneys.

Likewise, the experimental colitis seems to have caused activation of microglia or infiltration of macrophages in the brain, as *ex vivo* biodistribution studies revealed a small, but significant increase in [^11^C]PBR28 uptake in cerebellum 11 days after TNBS administration. In the previous studies, microglia activation in cerebellum has not been investigated, but it has been demonstrated in hippocampus at day 4 following TNBS treatment [32]. This effect, however, was not detected in our study; this could be caused by the difference in sensitivity of the PET tracer as compared to immunohistochemistry. In contrast to *ex vivo* biodistribution, repetitive [^11^C]PBR28 PET imaging could not reveal any activation of microglia or infiltration of peripheral macrophages in the brain as no significant differences in cerebral or cerebellar tracer uptake were observed between rats with colitis and controls.

## Conclusions

To summarize, interaction between intestinal inflammation associated with IBD pathology with the brain immune system may explain several brain-related comorbidities observed in patients with IBD. Longitudinal monitoring of inflammation in both the gut and the brain in animal models and in patients with colitis is necessary to confirm this hypothesis. For this purpose, it is essential to validate methods for reliable non-invasive measurement of inflammation that can be used both in animal studies and in clinical trials. In this study, we investigated the feasibility of monitoring brain and gut inflammation in a rat model of TNBS-induced colitis with [^11^C]PBR28 PET and organ harvesting. Intestinal inflammation, kidney infiltration, and mild neuroinflammation were detected in *ex vivo* biodistribution studies. However, these effects could not be detected by imaging in our colitis model due to an insufficient sensitivity of the technique. This may be caused by spill-over effects as a result of the limited resolution of the PET camera or by insufficient contrast to detect subtle changes in microglia/macrophage activation due to the fact that the background signal of [^11^C]PBR28 is still too high (although, it is lower than for many other TSPO tracers). On the other hand, the lack of detection of TSPO receptors with PET imaging could be caused by insufficient expression of these receptors. Although previous studies have demonstrated TSPO expression following TNBS injection [40], it is possible that more severe model with higher TNBS dose should have been applied here. This, however, still supports the hypothesis that PET imaging with [11C] is not a sensitive method for the detection of gut and brain inflammation in TNBS-induced colitis in rats.
